# Cancer Immunotherapy by Blocking Immune Checkpoints on Innate Lymphocytes

**DOI:** 10.3390/cancers12123504

**Published:** 2020-11-25

**Authors:** Silvia Pesce, Sara Trabanelli, Clara Di Vito, Marco Greppi, Valentina Obino, Fabio Guolo, Paola Minetto, Matteo Bozzo, Michela Calvi, Elisa Zaghi, Simona Candiani, Roberto Massimo Lemoli, Camilla Jandus, Domenico Mavilio, Emanuela Marcenaro

**Affiliations:** 1 Department of Experimental Medicine (DIMES) and Centre of Excellence for Biomedical Research (CEBR), University of Genova, 16132 Genova, Italy; silvia.pesce@unige.it (S.P.); s3735031@unige.it (M.G.); valentinaobino@gmail.com (V.O.); 2 Department of Pathology and Immunology, Faculty of Medicine, University of Geneva, CH-1211 Geneva, Switzerland; sara.trabanelli@unige.ch (S.T.); Camilla.Jandus@unige.ch (C.J.); 3 Ludwig Institute for Cancer Research, Lausanne Branch, CH-1066 Lausanne, Switzerland; 4 Unit of Clinical and Experimental Immunology, Humanitas Clinical and Research Center, 20089 Rozzano, Milan, Italy; clara.di_vito@humanitasresearch.it (C.D.V.); michela.calvi@humanitasresearch.it (M.C.); Elisa.Zaghi@humanitasresearch.it (E.Z.); domenico.mavilio@humanitas.it (D.M.); 5 Department of Medical Biotechnology and Translational Medicine (BIOMETRA), University of Milan, 20122 Milan, Italy; 6 Clinic of Hematology, Department of Internal Medicine (DIMI), University of Genoa, 16132 Genova, Italy; fabio.guolo@hsanmartino.it (F.G.); paola.minetto@hsanmartino.it (P.M.); roberto.lemoli@unige.it (R.M.L.); 7 IRCCS Ospedale Policlinico San Martino, 16132 Genova, Italy; 8 Department of Earth, Environment and Life Sciences (DISTAV), University of Genova, 16132 Genova, Italy; matteo.bozzo@edu.unige.it (M.B.); candiani@unige.it (S.C.)

**Keywords:** natural killer cells, innate lymphoid cells, immune checkpoint, immunotherapy, KIR, PD-1, NKG2A, innate immunity, immune escape, miRNA

## Abstract

**Simple Summary:**

The emergence of immunotherapy for cancer treatment bears considerable clinical promise. The role of NK cells in tumor immunosurveillance and their potential for successful cancer immunotherapy strategies is currently established. Specific focus is placed on the use of specialized monoclonal antibodies against NK cell immune checkpoints (ICI). The recent discovery that also helper ILCs express inhibitory IC suggests that these molecules might be also targeted on ILCs to modulate their functions in the tumor microenvironment. Herein, we provide an overview of the current knowledge on IC on NK cells and ILCs and we discuss how to target these innate lymphocytes by ICI in both solid tumors and hematological malignancies. Overall, we believe that, in our near future, immunotherapy protocols will need to be designed taking into account all ILCs, both cytotoxic (NK) and non-cytotoxic (helper ILCs) ones, and most importantly, ILCs targeting should be tailored according to the disease.

**Abstract:**

Immune checkpoints refer to a plethora of inhibitory pathways of the immune system that play a crucial role in maintaining self-tolerance and in tuning the duration and amplitude of physiological immune responses to minimize collateral tissue damages. The breakdown of this delicate balance leads to pathological conditions, including cancer. Indeed, tumor cells can develop multiple mechanisms to escape from immune system defense, including the activation of immune checkpoint pathways. The development of monoclonal antibodies, targeting inhibitory immune checkpoints, has provided an immense breakthrough in cancer therapy. Immune checkpoint inhibitors (ICI), initially developed to reverse functional exhaustion in T cells, recently emerged as important actors in natural killer (NK)-cell-based immunotherapy. Moreover, the discovery that also helper innate lymphoid cells (ILCs) express inhibitory immune checkpoints, suggests that these molecules might be targeted on ILCs, to modulate their functions in the tumor microenvironment. Recently, other strategies to achieve immune checkpoint blockade have been developed, including miRNA exploiting systems. Herein, we provide an overview of the current knowledge on inhibitory immune checkpoints on NK cells and ILCs and we discuss how to target these innate lymphocytes by ICI in both solid tumors and hematological malignancies.

## 1. Introduction

Innate lymphocytes (ILs) are a heterogeneous group of non-B and non-T lymphocytes. Recent progress in the understanding of their cytokine production, cytotoxic functions, and transcription factors involved in their development has led to an improved classification of these cells into five distinct subsets, namely natural killer (NK) cells, helper ILCs (i.e., ILC1s, ILC2s, ILC3s), and lymphoid tissue inducer (LTi) cells [1,2].

While NK cells represent the innate counterpart of CD8^+^ T lymphocytes, helper ILCs mirror the CD4^+^ T helper lymphocytes [3]. NK effector-functions are finely tuned by an array of receptors mediating either inhibitory or activating signals. The lack or reduced expression of self-human leukocyte antigen (HLA)-I alleles, a frequent event in malignant cells, induces the NK-cell activation and cytotoxicity through the release of perforin and granzymes as well as the rapid production of Interferon (IFN)-γ and tumor necrosis factor (TNF)-α [3,4,5,6].

In humans, NK cells can be further subdivided into functionally distinct subsets based on the surface expression of CD56 and CD16 that are differently distributed in healthy or inflamed tissue [4,7,8,9,10].

Helper ILC1s, similarly to NK cells and Th1 cells, express T-bet, produce IFN-γ, and represent potent effectors against infections, but are generally poorly cytotoxic [11,12].

Notably, a CD56^+^ILC1-like cell population with cytotoxic properties and sharing features with both ILC1s and CD56^bright^ NK cells has been recently described [13].

Similar to Th2 cells, ILC2s express high levels of GATA3 and are involved in the production of type 2 cytokines including interleukin (IL)-4, IL-5, IL-9, and IL-13, in response to IL-25 and IL-33 produced by epithelial cells or other immune cells following parasite infections or allergen exposure [2,12,14]. In addition, ILC2s produce amphiregulin, a member of the epidermal growth factor that helps repairing damaged tissues [15].

ILC3s are RORγt^+^ lymphocytes, mainly resident in the gut lamina propria, but also localized in skin, lung, liver, and decidua, where they participate in tissue homeostasis at the steady state and in protective immune responses against extracellular bacteria and fungi by secreting Th17 cytokines [16,17].

In addition to ILC3s, also LTi cells are able to produce IL-22 and IL-17 to initiate protective immune responses against extracellular bacteria [18]. They are mainly involved in lymphoid organogenesis during embryogenesis, but LTi-like cells have also been found in post-natal life, where they participate in the development of T and B cells [19].

Among the innate lymphocytes, NK cells are undeniably the best-studied mediators of anti-tumor innate immune responses. Indeed, NK cells are the most abundant innate lymphocytes and usually their presence correlates with a better prognosis and a decreased metastatic potential in human cancers [20,21]. Moreover, their ability to recognize and eliminate nascent transformed cells is corroborated by the observation that individuals with a decreased natural cytotoxic activity have an increased cancer risk [22].

In addition to NK cells, several lines of evidence indicate that helper ILCs are increased at the tumor site [23]. However, while NK cells possess a clear tumor-suppressive role in several cancers, helper ILCs are emerging to have pro- and anti-tumor properties depending on the microenvironment, probably because of their high plasticity and heterogeneity [24].

Furthermore, tumor cells can render all ILCs inefficient in controlling cancer initiation and progression, by developing multiple mechanisms to favor immune evasion. Among them, the activation of immune checkpoint (IC) pathways, through the expression of ligands for inhibitory receptors and the modulation of inhibitory and activating receptor expression, represents one of the most attractive targetable tumor-escape mechanisms to restore anti-tumor immunity [23].

As a matter of fact, the development of immune checkpoints inhibitors (ICIs), monoclonal antibodies (mAbs) targeting inhibitory receptors, has provided an immense breakthrough in cancer therapy. 

Herein, we review the current knowledge on inhibitory checkpoints expressed on NK cells and helper ILCs. Moreover, we provide a comprehensive overview on the recent insights to boost NK cells against cancer by using ICI, which can act directly, through the binding to the specific inhibitor receptor, or indirectly, by promoting antibody-dependent cell-mediated cytotoxicity (ADCC) (Table 1).

## 2. NK Cells

### 2.1. PD-1

PD-1 is an inhibitory receptor originally discovered on T cells and playing an important role in maintaining peripheral tolerance and T-cell homeostasis. However, its interaction with PD-ligands (PD-L1 and PD-L2), that may be expressed on tumor cells, can inhibit T-cell function, contributing to immune escape. For this reason, PD-1 has become one of the most investigated targets for cancer immunotherapy. Although most research has centered on inhibiting PD-1 on T cells, the interest in understanding its role also in NK cells is emerging.

Indeed, PD-1 is brightly expressed on a discrete subset of circulating and fully mature (KIR^+^NKG2A^−^CD57^+^) NK cells belonging to CD56^dim^ and (if present) to CD56^neg^ subsets, from one-fourth of healthy individuals (HDs) seropositive for human cytomegalovirus (HCMV). Importantly, the analysis at different time points of the PD-1^+^ cell subset in given individuals indicated that this population remains stable over time [26]. 

As a matter of fact, higher proportions of PD-1^+^ CD56^dim^ NK cells can be detected in patients affected by different tumors, including Kaposi sarcoma, peritoneal carcinomatosis (PC), and ovarian cancer (OC) [26,27,28,29,30]. Moreover, PD1^+^ NK cells have been found in Hodgkin lymphoma (HL), but its expression is mainly confined to the CD56^bright^ subset. The expression of PD-1 on NK cells has been also found to have a possible unfavorable prognostic role, being particularly increased in high-grade PC patients [31].

In addition to its surface expression, recent studies showed that human NK cells display an intracytoplasmic pool of PD-1-mRNA and PD-1-protein [48].

Of note, since the size of the PD-1^+^ NK cell subset is enriched in the tumor microenvironment, as compared to peripheral blood (PB) of the same patient, it is conceivable that, soluble factors and/or cells in the tumor microenvironment can induce PD-1 expression [26,49]. In this regard, a key role for HCMV in PD-1^+^ NK subset induction was suggested [26] and PD-1 expression in spleen NK cells was selectively induced by endogenous glucocorticoids in response to murine CMV infection [49]. Recently, a correlation between the presence of glucocorticoids together with high levels of pro-inflammatory cytokines (i.e., IL-12, IL-15, and IL-18) and *de novo* expression of PD-1 on CD56^bright^ NK cells has been established [50].

Importantly, the use of anti-PD-1 or anti-PD-L1 mAbs improves the anti-tumor activity of NK cells against PD-L1/2^+^ tumor cells [25,26,28,51]. This is clinically relevant for patients with tumors displaying a T-cell-resistant (i.e., HLA-I^−^) phenotype. In order to get an amplified and more effective response by both NK and T cells, several immunotherapeutic trials focused on the blockade of multiple ICs shared by these immune cells are ongoing (Table 1). In this regard, a combination of monalizumab (anti-NKG2A) and durvalumab (anti-PD-L1) has been evaluated in a first-in-human dose-escalation/dose-expansion phase I trial in patients with metastatic microsatellite-stable colorectal cancer (MSS-CRC). The rationale of this study was supported by preclinical models (https://www.innate-pharma.com/sites/default/files/180205asco_15poster_09.pdf) and was based on the hypothesis that the inhibition of NKG2A might improve the efficacy of PD-1/PD-L1-disrupting agents. This study included 40 patients in the MSS-CRC expansion cohort. The treatment was well-tolerated; 3 responses and 11 disease stabilizations were observed, with a disease control rate of 24% at 16 weeks [32].

### 2.2. KIRs

Killer immunoglobulin-like receptors (KIRs) can be divided into two categories depending on the number of extracellular Ig-like domains (two for the KIR2D and three for the KIR3D), and depending on the cytoplasmatic tail which dictates the function of the molecule into: Inhibitory KIRs (iKIR), with a long (L) cytoplasmic tail with two tyrosine-based inhibitory motifs (ITIMs); activating KIRs (aKIR), with a short (S) cytoplasmic tail containing a charged amino acidic residue associated to the KARAP/DAP12 adaptor molecule, bearing immunoreceptor tyrosine-based activating motifs [52,53]. In humans, 13 genes and 2 pseudogenes coding for KIR molecules have been identified. An additional step of KIR heterogeneity is given by the high number of polymorphisms of these molecules (1110 different KIR polymorphisms currently identified in the IPD-KIR Database, release 2.9.0).

KIRs are clonally expressed on NK cells, meaning that each cell expresses a different set of KIRs, determined randomly. Only cells expressing at least one KIR (or the heterodimer CD94/NKG2A) that recognizes self-HLA undergo “education” and become licensed [54]. Indeed, the higher the binding of iKIRs to their ligands during NK-cell maturation is, the higher the cytotoxicity of the cell is. Conversely, a high binding of aKIRs to their ligands leads to a lower cytotoxicity [55]. Generally, NK cells recognize and kill cells that do not express or express low levels of ligands for their iKIRs. This mechanism is defined as “missing self-hypothesis” and it is the reason why NK cells are fundamental in tumor immunosurveillance.

Of note, the interaction between KIR and HLA-I may act as promoter (aKIR) or dampen (iKIR) for a phenotype change. In particular, the highly cytotoxic CD56^dim^ KIR^+^ NK cells, can acquire surface CCR7 upon interaction with CCR7^+^ cells, becoming able to migrate in response to the secondary lymphoid-tissue chemokines CCL19/CCL21. This novel NK-cell ability occurs through a trogocytosis mechanism and precedes the NK-mediated cytolysis [56,57,58,59]. 

Specifically, NK cells are fundamental for recognizing tumors downregulating HLA-I molecules in order to escape from T cells [53] Reversely, for tumor cells that do not loose HLA-I, iKIRs can be considered as additional IC that aid the immunoevasion of the transformed cells. For this reason, numerous immunotherapies based on mAbs blocking iKIR-HLA interactions have been developed to unleash NK cells against HLA-I^+^ tumor cells, thus increasing the potential activity of these cytotoxic innate effectors (Table 1).

The first anti-pan-KIR2D developed, lirilumab (Innate Pharma) [34], is included in at least thirteen clinical trials (4 Phase-I, 5 Phase-II, 4 Phase-I/II) with two studies terminated [both Phase II trials, one was terminated because of the sponsor’s decision not to pursue the development of lirilumab for myeloid malignancies (NCT02599649), the other was terminated because of the response rates not meeting the anticipated minimum of 30% (NCT02399917)]. Of these studies six are on solid tumors, six on hematological malignancies and one is on all pediatric tumors. Although lirilumab is well tolerated for doses up to 10 mg/kg in monotherapy, unfortunately monotherapy with lirilumab did not show significative results, thus its effects are now mostly studied in combination with other ICIs, especially nivolumab (anti-PD-1, 7 clinical trials). A clinical trial, completed on 11 August 2020, studying the effect of lirilumab in combination with nivolumab and with nivolumab and ipilimumab (anti-CTLA-4) in patients with advanced solid tumors, was presented in a press release. The combination of lirilumab and nivolumab in squamous cell carcinoma of the head and the neck (SCCHN) patients (*n* = 29) was well-tolerated and resulted in an objective response rate of 24%, with durable response (NCT03341936).

Lirilumab is currently under study also in combination with elotuzumab (anti-CS1, NCT02252263) or rituximab (anti-CD20, NCT02481297) in multiple myeloma (MM), and with nivolumab or lirilumab and ipilimumab, together with other drug combinations, in MM, HL, and non-Hodgkin (NHL) lymphomas (Phase I/II trial, NCT01592370).

In addition to mAbs targeting KIRs, alternative strategies to target the KIR/HLA-I axis are under investigation. As an example, a miRNA (miR146a-5p) targeting KIR2DL1/KIR2DL2 mRNA by abrogating their expression has been recently identified [60,61]. This result may be exploited to generate/increment the effect of NK-cell KIR-mismatching against HLA-I^+^ tumor cells and thus improve the NK-mediated anti-tumor activity.

### 2.3. NKG2A

NKG2A is an inhibitory receptor that, together with its activating counterpart NKG2C, belongs to the C-type lectin receptor family. It is expressed in association with CD94 on almost 50% of the NK cells in the PB [62].

The CD94/NKG2 complexes recognize the non-classical HLA-E molecule, expressed in most human tissues and displaying signal peptides derived from leader peptide sequences of other HLA-I molecules [63,64] or HCMV [65]. The engagement of CD94/NKG2A leads to the phosphorylation of their ITIM, resulting in an inhibitory signal that suppresses competing signals from NKG2C [63,66,67]. Under physiological conditions, this mechanism provides an important “self-signal” to allow self-tolerance and to prevent the destruction of self-bystander cells.

In human cancer, while classical HLA alleles are frequently lost to prevent T-cell recognition [68], HLA-E expression is even upregulated, as a protective mechanism of cancer cells against immune surveillance and elimination by cytotoxic lymphocytes [69]. Indeed, IFN-γ secreted by immune cells during anti-tumor immune responses upregulates the expression of HLA-E on tumor cells [70,71]. High levels of HLA-E have been reported in several cancer types, including NSCLC [72], glioblastoma [73], melanoma, breast (BC) [74], liver [75], kidney [76], gynecological [77], and colorectal cancers [78,79].

In turn, the overexpression of HLA-E on cancer cells drives the upregulation of CD94/NKG2A on cytotoxic lymphocytes, including NK cells, in both hematological and solid tumors [80]. In fact, tumor-infiltrating NK cells have higher expression of NKG2A than non-tumoral NK cells in NSCLC [81,82], BC [83], and OC [84]. Moreover, a direct correlation between levels of HLA-E expression and those of NKG2A and CD94 in tumor-infiltrating lymphocytes has been observed [80].

The binding of CD94/NKG2A to HLA-E/peptide on tumor cells results in the inhibition of the effector-functions of NK cells [85] and of other cytotoxic lymphocytes, thus leading to a poor prognosis in various solid cancers [74,78]. Likewise, phenotypic abnormalities have been reported also in NK cells from patients affected by hematologic malignancies. Indeed, PB NK cells from acute myeloid leukemia (AML) patients show impaired effector functions and an upregulation of NKG2A accompanied by decreased expression of NKp46 [86]. 

These data highlight an important role of the NKG2A/HLA-E axis in preventing the activation of cytotoxic NK cells in the tumor microenvironment, thus making NKG2A a suitable targetable checkpoint to unleash NK-cell-mediated responses against HLA-E^+^ tumor cells.

As a matter of fact, a mAb against NKG2A (IPH2201, monalizumab) has been developed by Innate Pharma (Marseille, France) in partnership with AstraZeneca (Cambridge United Kingdom) [36] and it is now employed in various trials for the treatment of different tumors (reviewed in [87]). In this regard, a phase I clinical trial (NCT02459301) in patients with advanced, recurrent, or metastatic gynaecologic cancers has been completed and shows that monalizumab administration leads to short-term stabilization with minimal treatment toxicities and excellent treatment tolerance [35].

Moreover, since preclinical studies showed that anti-NKG2A in combination with anti-PD-L1 mAbs has a synergistic effect on tumors expressing both HLA-E and PD-L1 [36], several clinical trials are now investigating the effectiveness of monalizumab in combination with other compounds, including anti-PD-L1 (NCT02643550, NCT03822351, NCT04145193, NCT03833440, NCT02671435, NCT03794544), anti-EGFR (NCT02643550), and anti-Bruton’s tyrosine kinase (NCT02557516) [5,88] (Table 1).

In the context of hematologic malignancies, we recently described in patients undergoing a haploidentical hematopoietic stem cell transplantation (haplo-HSCT) the early expansion of anergic donor-derived NK-cell subpopulation expressing high levels of NKG2A [10]. Ongoing studies are now investigating whether the administration of monalizumab, early after haplo-HSCT, can restore the NK-cell cytotoxicity thus improving engraftment and limiting the onset of opportunistic infections and the occurrence of acute graft versus host disease [87].

In addition to mAbs targeting NKG2A, alternative strategies to target the NKG2A/HLA-E axis are under investigation. As an example, a construct containing a fragment derived from an anti-NKG2A antibody linked to endoplasmic reticulum-retention domains, has been developed to abrogate NKG2A expression [80].

### 2.4. TIM-3

T-cell immunoglobulin and mucin domain 3 (TIM-3) is an inhibitory receptor able to recognize several ligands. In particular, TIM-3 binds galectin-9, which is upregulated in various cancers and chronic infections [89,90,91,92,93,94], causing the apoptosis of Th1 cells [95]. The TIM-3 variable IgV domain has also been reported to bind high mobility group protein B1 and this binding compromises the activation of immune responses. In addition, TIM-3 recognizes phosphatidylserine (PtdSer) [89,90] and carcinoembryonic antigen cell adhesionmolecule 1 (Ceacam-1). PtdSer is over-expressed in apoptotic cells and induces the clearing of apoptotic bodies and the reduction of antigen cross-presentation by dendritic cells (DCs) [74,96], while the interaction between TIM-3 and Ceacam-1 is involved in T-cell exhaustion promotion, thus suggesting that the inhibitory function of TIM-3 depends on Ceacam-1 co-expression [97].

TIM-3 expression is quite spread among immune cells, indeed CD4^+^, CD8^+^ and regulatory (Treg) T cells, B cells, NK cells, NKT cells, and myeloid cells may be TIM-3^+^ [90,98]. TIM-3 engagement with its ligands induces immune tolerance by exhausting T cells as well as NK cells [39,92,99,100].

Regarding NK cells, TIM-3 is highly expressed in resting NK cells as compared to the CD56^+^ NKT and CD56^+^ T cells [101]. Its expression is mainly restricted to the CD56^dim^ subset and may be upregulated on CD56^bright^ cells upon cytokine stimulation [101,102]. High frequencies of circulating and/or tumor infiltrating TIM-3^+^ NK cells have been found in different types of malignant tumors [38,40,103] including gastric cancer (GC) [38], lung adenocarcinoma [39], advanced melanoma [40], and bladder cancer [38,39,40,41]. Importantly, while the increased surface levels of TIM-3 on NK cells in cancers induce NK-cell impairment [41], the in vitro and ex vivo TIM-3 blockade results in increased NK-cell cytotoxicity [39,40,104]. Contrariwise, studies have also reported stimulatory functions of TIM-3 [105]. These divergent functions are likely associated with the existence of multiple and different TIM-3 ligands.

Co-expression of TIM-3 and PD-1 was shown to mediate the exhaustion of CD8^+^ T cells in various cancers and chronic viral infections [106,107,108,109,110]. On the contrary, clear data about a possible co-expression of TIM-3 and PD-1 on NK cells are not yet available. In this context, anti-TIM3 antibodies, including Sym023, cobolimab, LY3321367, BGB-A425, and MBG453, in combination with several anti-PD-1/PD-L1 antibodies, are under clinical investigation for their efficacy against various cancers. Sym023 has been developed and is being tested in phase I clinical trials, in patients with advanced and metastatic solid tumor malignancies or lymphomas refractory to currently available therapies, in monotherapy or in combination with anti-PD-1 or anti-LAG-3 antibodies (NCT03489343 and NCT03311412). Additional phase I studies of anti-TIM-3 antibodies have been initiated in patients with advanced solid tumors, as a monotherapy or in combination with an anti-PD-1 antibody (NCT02817633, NCT03680508, NCT04139902, NCT03680508, NCT03744468). Relatlimab (BMS-986016 TSR-022, Tesaro Inc, Waltham, MA, United States) is under investigation in three clinical trials in advanced cancer alone or in combination with anti-PD-1 (NCT02817633, NCT03307785), and in primary liver cancer in combination with anti-PD-1 (NCT03680508). MBG453 (Novartis) is being evaluated as monotherapy or in combination with anti-PD-1 in patients with advanced malignancies (NCT02608268) and patients with AML or high-risk myelodysplastic syndromes (NCT03066648) (Table 1).

### 2.5. TIGIT

T-cell immunoreceptor with immunoglobulin and ITIM domains (TIGIT) is an immune inhibitory receptor of T and activated NK cells. TIGIT competes with the activating receptor DNAM-1 for binding PVR and Nectin-2 [111]. Upregulation of both TIGIT and its ligands have been described in multiple cancer types. TIGIT expression on NK cells, and the consequent exhaustion of these cells, has recently been described in colon cancer patients and tumor models [42]. Moreover, the expression of PVR was shown to correlate with a diminished tumor infiltration and with increased VEGF expression and increased angiogenesis [112]. TIGIT expression was proven to suppress both NK and CD8^+^ T-cell function in CRC growth [42].

Conversely to CTLA-4 and PD-1, TIGIT was associated to NK-cell exhaustion in a mouse model of colon cancer and TIGIT blockade was capable of reverting the condition allowing NK cells to trigger an anti-tumor immune response. TIGIT blockade was proven to synergize with anti-PD-L1 antibody therapy helping memory immunity in tumor rechallenge models [42]. 

In hematological tumors, TIGIT was suggested as a biomarker in the context of allogeneic HSCT, because of its correlation with a diminished NK-cell count in the bone marrow (BM) after transplant. Thus, TIGIT blockade was suggested as a potent strategy to increase graft versus leukemia effect in allogeneic HSCT in AML [43].

Because of the rapid consolidation of TIGIT as an important player in immuno-escape, numerous anti-TIGIT mAbs are currently being tested for clinical use. In this regard, tiragolumab (Genetech/Roche, South San Francisco, CA, United States) is currently under investigation together with atezolizumab (anti-PD-1) on cervical cancer (NCT04300647), esophageal squamous cell carcinoma (NCT04543617), small cell lung cancer (NCT04256421), NSCLC (NCT04513925, NCT03563716), esophageal cancer (NCT04540211, NCT03281369), advanced liver cancer (together with the anti-VEGF bevacizumab, NCT04524871), urothelial carcinoma (NCT03869190), pancreatic adenocarcinoma (NCT03193190), and advanced metastatic tumors (NCT02794571). Moreover, BMS-986207 (Bristol-Myers Squibb, New York, NY, United States) is being tested as a single agent in MM (NCT04150965) and as a single agent or together with nivolumab on advanced solid tumors (NCT04570839).

Etigilimab (OncoMed Pharmaceuticals, Redwood City, CA, United States) was tested together with nivolumab in a single trial that was terminated because of the decision of the sponsor (NCT03119428); AB154 (Arcus Bioscience, Hayward, CA, United States ) is being tested alone and together with zimberelimab (anti-PD-1) in advanced solid tumors (NCT03628677) and in lung cancer (NCT04262856) (Table 1).

### 2.6. LAG-3

Lymphocyte activation gene-3 (LAG-3) is a member of the immunoglobulin superfamily receptors with inhibitory properties [113]. LAG-3 is a negative co-inhibitory receptor mainly expressed on T and NK cells, but also on other immune cells including tumor infiltrating lymphocytes (TILs), Treg, iNKT cells, B cells, and DCs [89,114,115,116,117,118,119]. It recognizes MHC class II (MHC-II) molecules (with greater affinity than CD4) [113,120,121], the C-type lectin receptor LSECtin, and a fibrinogen-like protein1 (FGL1) on target cells [120,122,123].

Engagement of LAG-3 promotes T-cell exhaustion [124,125,126] and the suppressive activity of Treg [116,127]. In addition, LAG-3 synergizes with PD-1 in T-cell functional regulation to promote tumor immune escape [128]. LAG-3 has been shown to suppress immune responses in several tumors, including HL, GC, BC [111] and its blockade restores T-cell functions [125]. On the other hand, its specific role in regulating NK-cell function is still not fully clarified and recent findings suggest that LAG-3 is expressed on “adaptive” NK cells chronically exposed to HCMV rather than on activated NK cells [129,130].

Nevertheless, LAG-3 is currently considered a good target for immunotherapy in order to strengthen not only T-cell anti-tumor activity, but also the NK cell once probably through ADCC.

In this context, different anti-LAG-3 antibodies [i.e., relatlimab (BMS-986016)] are currently being used in phase I and phase II clinical trials as single drugs in metastatic cancers, solid tumors and lymphoma (NCT03489369), or in association with other ICIs, including, anti-TIGIT in MM (NCT04150965), with or without anti-PD-1 antibodies in treating patients with advanced NSLCC (NCT02658981), solid tumors (NCT01968109, NCT03470922, NCT02720068, NCT03250832, NCT02460224, NCT03365791), and hematologic malignancies (NCT03365791, NCT03598608), advanced malignancies (NCT03005782) including SCCHN (NCT04080804), triple-negative BC (NCT03499899), and metastatic melanoma (NCT03484923). A number of additional LAG-3 antibodies are currently in preclinical development (Table 1).

In addition, a soluble recombinant LAG-3-Ig fusion protein, Eftilagimod alpha (IMP321), has been used as an immunological adjuvant for vaccination against various infections and cancer. It has been employed as monotherapy or combined with chemotherapy in cancer (NCT02676869) [45], in metastatic BC (NCT00349934), in patients with melanoma (NCT01308294) and with PD-1 in SCCHN (NCT03625323). 

IMP321 was able to induce cytokine production (IFN-γ and/or TNF-α) by NK cells in PBMCs from HDs and, to a lower extent, in PBMCs from 21 untreated metastatic cancer patients in a short-term ex vivo assay [131]. In metastatic renal cancer patients, IMP321, in a dose-escalation study (P003), induced NK-cell activation as monotherapy [132]. In BC patients IMP321, associated with standard chemotherapy, induced an enhanced NK-cell activation for several months [133].

Hence, LAG-3 has the potential to activate T cells as well as NK cells and it can be further explored as a potential target for checkpoint inhibition.

## 3. Helper ILCs

NK cells being the innate counterparts of CD8^+^ T cells, helper ILCs are viewed as mirrors of CD4^+^ T helper cells. Despite many shared patterns, we and others also identified distinct regulatory circuits of ILC and T helper by comparing their transcriptomic profiles [134,135,136,137]. Of note, transcripts encoding for ICs are less abundant in human resting ILCs than in CD4^+^ T-helper cells, eventually contributing to their ability to swiftly respond to external stimuli in an antigen-independent manner. Upon activation, a dynamic modulation of inhibitory receptor expression occurs to ensure proper termination of the response and resolution of inflammation. However, while the role of checkpoint molecules has been largely explored in T-cell responses, only recent studies have started to uncover the involvement of ICs in tuning helper ILC-dependent immunity, including in anti-tumor responses.

### 3.1. PD-1

PD-1 was initially described as a marker of ILC progenitors [137,138]. Single-cell RNA-seq analysis on Lin^-^Flt3^lo/−^IL-7Rα^lo/+^α_4_β_7_^+^ BM cells revealed a cluster of ILCs expressing *Pdcd1* (encoding for PD-1) together with *Id2*, *Tcf7*, *Tox*, and *Gata3*. Differently from T cells, the ILCs belonging to this cluster did not express other inhibitory or activation molecules. Isolated PD-1^hi^ ILCs, transferred in vivo or individually seeded in vitro, differentiated in all the ILC subsets, without generating B or T cells, demonstrate that PD-1 expression identified unipotent and pluripotent ILC progenitors. A deeper analysis showed that only the PD-1^hi^ ILCs, also positive for *Bcl11b* and IL-25R, were able to differentiate exclusively into ILC2s, defining IL-25R as a marker of early ILC2 progenitors in PD-1^hi^ cells. However, also a certain percentage of peripheral terminally differentiated ILCs expressed PD-1 and, upon influenza infection or papain challenge, proliferated and secreted high amount of IL-13 and IL-5. On the contrary, Taylor et al. [139] showed that PD-1^+^KLRG1^+^ ILC2s had a proliferative disadvantage in comparison to PD-1^−^KLRG1^+^ ILC2s. This result, however, was not confirmed by Batyrova et al. [140], who reported no difference in KLRG1^+^ ILC2s between *Rag1^−/−^* and *Pdcd1*^−/−^ *xRag1^−/−^* mice. 

Some differences in the experimental models could explain the dichotomies observed in these first publications. First of all, Taylor et al. [139] used *Pdcd1*^−/−^ mice that were not used by Yu et al. [137], and Batyrova et al. [140]. used *Rag1^−/−^* as background for all the experiments. Taylor et al. [139] and Batyrova et al. [140], moreover, focused on KLRG1^+^ ILC2s and helminth infection, differently from Yu et al. [137], that were not restricting their analysis to the ILC2s expressing KLRG1 and that were using influenza infection and papain challenge. In addition, while Taylor et al. [139] identified in IL-33 the trigger for PD-1 expression and Batyrova et al. [140] in PPAR-γ agonists, Yu et al. [137], did not determine the signals inducing the PD-1^+^ ILC progenitor. Another interesting point is that, in the model of Taylor, after 3 days of stimulation, ILC2s started to upregulate PD-L1. It is therefore possible that the PD-1/PD-L1 interaction on different ILC2s could influence their proliferation and function leading to different results overtime. 

PD-1 expression on ILC2s is modulated by cytokines. A cascade involving TNFα-induced IL-33 secretion by pre-adipocytes was shown to induce PD-1 expression in adipose tissue ILC2s in obese mice. As a consequence, PD-L1^high^M1 macrophages triggered PD-1 on ILC2s in models of high-fat diet-induced obesity, leading to ILC2 dysfunction and limiting type-2 mediated tissue beiging. Therapeutic blockade of PD-1 in these animals partially restored ILC2 functions and ameliorated the metabolic homeostasis [141]. In line with these findings, Helou et al. [142] reported that PD-1 is inducible on pulmonary ILC2s upon intranasal IL-33 stimulation. By comparing the transcriptomic profiles of pulmonary ILC2s from WT and PD-1 knockout mice the authors highlight an in cis interaction between PD-1 and PD-L1 in IL-33 activated ILC2s. This binding, as well as an in trans PD-1/PD-L2 engagement, is responsible for the impaired ILC2 cytokine secretion and survival that mechanistically occurs at least in part through a metabolic switch. Indeed, deficiency of PD-1 resulted in the upregulation of glycolysis-dependent genes and in the increase of amino acid degradation, showing for the first time a PD-1-dependent metabolic inhibition of ILC2 proliferation. By developing and testing a PD-1 agonist in a humanized mouse model of house dust mite-induced asthma the authors demonstrate the potential of PD-1 inhibition of ILC2s in asthma treatment.

Beside ILC2s, PD-1 is also expressed by human group 3 ILCs in the decidua [17]. During the first trimester of pregnancy, CD56^+^ ILC3s and CD56^−^ ILC3s (also referred as lymphoid tissue inducer (LTi-like cells) expressed PD-1 and TIM-3 (see also below), but not LAG-3 and TIGIT. PD-1 expression limited the ILC3 cytokine production and decreased after the first trimester. Because intermediate extravillous trophoblast (iEVT) and decidual stromal cells express PD-L1, the presence of PD-1^+^ ILC3s during the first trimester could contribute to the feto-maternal tolerance. This speculation is supported by the fact that, in samples obtained from spontaneous abortion, PD-L1 expression in iEVT was much lower than in normal pregnancies. However, this new mechanism of immune-tolerance would need to be confirmed and analyzed in in vivo models.

### 3.2. PD-1 Expression on ILCs in Cancer 

Salimi et al. were the first in documenting the expression of ICs (i.e., PD-1 and CTLA-4) in ILCs present in human tumor tissues [24]. They observed no difference in the expression of PD-1 in ILCs present in malignant versus benign BC tissues. However, the level of PD-1 in tumor-associated ILC2s was higher in comparison with the circulating ILCs. Interestingly, in gastrointestinal tumors, ILC2s and ILC3s express higher PD-1 than in the perilesional tissue. This phenotype was partially associated with an increase in HLA-DR, KLRG1, CD69, and CD44, and with a decrease in CCR7 expression. Whether PD-1^+^ILCs in tumor tissues are more activated or exhausted is yet to be determined.

Subsequently, the analysis of the innate cell compartment of patients presenting human-malignant pleural effusion revealed an infiltration by all the ILC subsets, with a prevalence of PD-1^+^ ILC3s [29]. By activating these cells through their natural cytotoxicity receptors (NCR, i.e., NKp30, NKp44 and NKp46), the authors showed that a small proportion (2.5–6% of NCR^+^ ILC3s) could produce IFN-γ and TNF-α. Interestingly, by using an anti-PD-1 antibody or a recombinant PD-L1 molecule, the production of IFN-γ and TNF-α was inhibited, suggesting that, in the tumor microenvironment, the anti-tumor activity of ILC3s could be repressed because of their PD-1 expression. 

In another work, Wang et al. identified six ILC subsets by performing single-cell RNA sequencing to profile tumor-infiltrating ILCs in an azoxymethane/dextran sodium sulfate (AOM/DSS)-induced colitis-associated colorectal cancer (CRC) model [33]. Among those, three subsets expressed ILC2 signature genes (ILC2-A, -B, and -C). The ILC2-C subpopulation expressed high PD-1 and HS3ST1 (heparan sulfate3-O-sulfotransferase 1). Because of several elegant experiments, among which are the transfer of PD-1^high^ILC2s in B-NSG mice, the deletion of PD-1 and HS3ST1 from ILC2s by CRISPR-Cas9 technology, the generation of *Hs3st1*^flox/flox^*Id2*-CreERT2 mice, the targeting of PD-1 with an anti-PD-1 antibody, Wang et al. demonstrated that PD-1^+^ and HS3ST1^+^ ILC2s are involved in CRC tumor progression.

Further, Moral and colleagues dissected a synergistic effect of anti-PD1 blockade targeting both anti-tumor ILC2s and CD8^+^ T cells in pancreatic ductal adenocarcinoma (PDAC) [143]. The authors reported on abundant infiltration of human and murine PDAC by ILC2s. In mice, these cells were induced by in situ-secreted IL-33 to release CCL5, recruiting CD103^+^ DCs that in turn promoted the priming of cytotoxic CD8^+^ T cells. Of note, as previously reported by others, IL-33 induced PD-1 expression on ILC2s, and combined administration of recombinant IL-33 and anti-PD-1 antibodies acted synergistically in sustaining ILC2 anti-tumor functions and in restoring CD8^+^ T-cell cytotoxicity. This resulted in significant tumor size reduction and prolonged animal survival. As a correlate, PD-1^+^ ILC2s were detected in human PDAC and IL-33 mRNA transcripts correlated with improved survival in patients with PDAC (Table 1).

Further work is needed to decipher the implication of PD-1 in pro- or anti-tumor functions of ILC2s in cancer and to define the impact of anti-PD-1 immunotherapy on ILC2s.

### 3.3. CTLA-4

In steady state conditions, CTLA-4 transcripts are poorly present in ILCs and cell surface protein is almost undetectable in human ILCs [135]. Salimi et al. reported the expression of CTLA-4 in tumor-associated ILC1s and ILC2s, being higher in comparison with the circulating ILCs, while no difference was observed in malignant versus benign BC tissues [24]. Gao and colleagues observed in different murine tumor models higher expression of CTLA-4 in intermediate ILC1s (intILC1s, being CD49a^+^CD49b^+^EOMES^+^), that are impaired in their ability to secrete IFN-γ but not TNF-α [144] (Table 1). The excess of TNF-α and the secretion of VEGF by ILC1s might be linked to pro-tumoral and pro-angiogenic phenomena. However, the mechanism(s) of regulation of cytokine secretion by CTLA-4 in ILCs remains to be elucidated.

### 3.4. TIGIT and CD96, and TGF-β

Beside their expression on conventional NK cells, TIGIT and CD96 are also found on ILC1s and splenic ILC3s [46]. While TIGIT targeting on NK cells, either alone or in combination with other ICs, leads to the restoration of anti-tumor activities [42], the functional consequence of TIGIT and/or CD96 blockade on ILCs remains unknown. Using different tumor mouse models, Gao et al. [144] reported that the intratumoral TGF-β mediates the conversion of anti-cancer NK cells into intermediate ILC1s (CD49a^+^CD49b^+^Eomes^+^) and ILC1s (CD49a^+^CD49b^−^Eomes^int^). This shift was accompanied by an upregulation of TIGIT and CD96, and concomitant reduction in DNAM-1 levels, thus creating a pro-tumor environment [144]. Besides its effect on NK-ILC1 plasticity, TGF-β signaling was also reported to drive ILC3 conversion into ILCs with a regulatory phenotype [33]. Indeed, Wang et al. [33] showed that during CRC progression, the proportion of ILC3s was decreased by concomitant expansion of Id3^+^ IL-10 secreting cells. Using either *Tgfbr2*^flox/flox^*Id2*-CreERT2 mice to delete the TGF-β receptor on ILCs or a TGF-β inhibitor, the conversion was inhibited and the tumor growth suppressed. This data suggested that the interference with the TGF-β signaling could be considered as a targetable checkpoint to fire at tumor infiltrating ILCs [44] (Table 1).

### 3.5. KLRG1

The C-type lectin KLRG1 is expressed by a subset of ILC2s, in vivo expanded by IL-25 administration, involved in anti-helminth responses and called “inflammatory” ILC2s [145]. KLRG1 ligation to E-Cadherin in vitro leads to ILC2 functional impairment with decreased IL-5 and IL-13 secretion [47], while its in vivo relevance remains unexplored. Interestingly, KLRG1 was also found to be expressed in a small percentage in ILC3s present in lymphocytes isolated from first-trimester decidual tissues [17] (Table 1). However, the role of these KLRG1^+^ ILC3s has not been elucidated yet. In cancer, KLRG1 transcripts have been identified in ILC2s in lung and CRC [20].

### 3.6. TIM-3

TIM-3^+^ human ILC3s were found in the decidua, to be inhibited in their IL-22 production upon TIM-3 cross-linking [17] (Table 1). Differently from PD1^+^ ILC3s, TIM-3^+^ ILC3s did not decrease after the first-trimester of pregnancy. Also in this case, in vivo experiments in animal models could provide more insights into the role of TIM-3^+^ ILC3s present at the feto-maternal interface. Nothing is known to date on the role of this type 1 glycoprotein on ILCs in cancer settings.

### 3.7. LAG-3

Expression of LAG-3 in resting ILCs has not been reported yet. As compared to their adaptive counterparts Th17 cells, that express LAG-3 to some extent, ILC3 were reported to be LAG-3 negative [135]. However, ILC3 are involved in a 3-cell party regulation dependent on LAG-3^+^ Tregs. In that axis, LAG-3^+^ Tregs, interacting with MHC-II on CX3CR1^+^ intestinal tissue-resident macrophages, restrained their secretion of IL-23 and IL-1β. As a consequence, the lack of a supportive environment reduced ILC3 activation and secretion of IL-22, thus suppressing colitis [146].

Moreover, Gao et al. reported that upon conversion from NKs to ILC1s in tumors, several checkpoints were upregulated, among them LAG-3, reaching much higher levels than on conventional NKs [144] (Table 1).

### 3.8. NKG2A

Another checkpoint molecule expressed by ILCs is NKG2A. We showed that, in humans, a population of Lin^-^CD127^+^CD56^+^c-Kit^−^ ILC1-like ILCs is present in HD and in AML patients [37]. In HDs, these cells are able to kill tumor cells in a KIR-independent manner, while in AML this ability was impaired. The ILC1-like ILCs isolated from AML patients expressed lower levels of TRAIL, NKp30, and NKp80 than the ones from HD. However, they expressed high levels of the CD94/NKG2A heterodimer (up to 80%) that, upon engagement with HLA-E on leukemic targets, resulted in decreased degranulation and impairment of ILC cytotoxic functions. Whether targeting NKG2A in AML patients with monalizumab would result in a more efficient killing of the leukemic target by the ILC1-like ILCs is still to be determined.

Additionally, along with the induction of LAG-3, KLRG1, and CTLA-4 on TGF-β-induced ILC1s, NKG2A is also overexpressed during the conversion from NK cells. Yet, its functionality has not been investigated, but decreased functionality of converted ILC1s is most likely due to checkpoint upregulation [144]. However, it remains to be determined if these cells express the CD94/NKG2A heterodimer able to deliver inhibitory signals upon HLA-E engagement, similarly to NK cells.

### 3.9. Metabolic Checkpoints

Beside the conventional IC presented above, a novel ILC2-dependent metabolic immune checkpoints has recently been uncovered [147]. The authors show that pre-existing type2 inflammation in the lung favors metastatic seeding, in an IL-33 and ILC2-dependent manner. ILC2s were driving the local IL-5-dependent eosinophil accumulation that correlated with impaired NK-cell activities. Mechanistically, eosinophils can suppress NK-cell functions by extracellular glucose deprivation and lactic acid increase, thus impairing the NK-cell glycolysis-dependent effector functions. In this context it has been shown that the NK/eosinophil cross-talk is regulated by the interaction of NCRs with eosinophil surface ligands [148]. Future work is needed to determine how to target these novel metabolic checkpoints to reinvigorate anti-tumor immunity, either alone or in combination with the conventional ICI.

## 4. Conclusions

It is now evident that ICI have provided an historical step forward in our multi-year effort to fight oncological diseases. Pioneer studies showed T cells as the main actors of these successes, but it is now clear that also NK cells may represent an excellent cancer immunotherapy tool because of their ability to kill malignant cells without toxicity toward healthy cells. This is clinically relevant for patients with tumors displaying a T-cell-resistant (HLA-Ineg) phenotype. In this context, the ICI specific for NK cells, such as monalizumab and lirilumab, aimed at NKG2A and KIR inhibitory receptors respectively, have been assessed as monotherapy, and have shown good safety profiles, but mild success in terms of prolonging progression-free survival. This result is not completely surprising, because of the complexity of immuno-regulatory mechanisms and the heterogeneity of malignancies. Thus, future clinical trials will have to reveal the potency of these NK-cell ICI in combination with other cancer treatment options. Importantly, the combinations of ICI, such as CTLA-4 and PD-1 inhibitors, that are being tried for synergistic response targeting T cells could also be tried in the context of NK cells. In this regard, it has recently been shown that PD-1 receptor is not only expressed by NK cells, but it has been also demonstrated that PD-1 and PD-L1 inhibitors are able to enhance NK-cell-mediated cytotoxicity [26]. Similarly, the anti-NKG2A mAb monalizumab can boost CD8^+^ T-cell as well as NK-cell immunity. Hence, a combination of a PD-1 or PD-L1 inhibitor and a NK-cell-specific checkpoint inhibitor (such as an anti-KIR or anti-NKG2A mAb), could be of value for combined checkpoint inhibition-based immunotherapy. Thus, an eye should be kept on the ongoing clinical trials exploiting this kind of new combinations.

It is also worth considering that despite all steps forward, immune checkpoint blockade therapies are not always effective, and even responding patients may subsequently relapse. Thus, the need of a deeper monitoring of cytotoxic lymphocytes (T and NK cells) in the tumor microenvironment and of their IC expression and interplay is needed to develop more effective treatments. In this regard, another strategy might be to call in our aid helper ILCs. Indeed, while the role of checkpoint molecules has been largely explored in cytotoxic lymphocytes, only recently studies have started to uncover the involvement of immune checkpoints in fine-tuning ILC-dependent immunity. In this context, several studies show that IC molecules may play an important role also in ILCs in anti-tumor responses. Although, as of today, there are no ILC therapies ongoing, we already know that targeting ILCs in AML using monalizumab would be beneficial to unleash the cytotoxicity of ILC1-like cells. Based on the pre-clinical observations of Moral et al. [143], PD-1 targeting on ILC2s might be beneficial in pancreas adenocarcinoma. Similarly, this strategy might activate ILC3s, as suggested by Tumino et al. [29]. In contrast to that, this same therapeutic strategy might be detrimental in settings where ILC2s exert pro-tumoral functions. Indeed, the administration of anti-PD-1 might favor ILC2 activity and increase the secretion of pro-tumoral Type-2 cytokines, supporting tumor growth [144]. Thus, additional studies on checkpoint expression in ILCs are needed to define optimal ILC targeting strategy.

**Figure 1:**
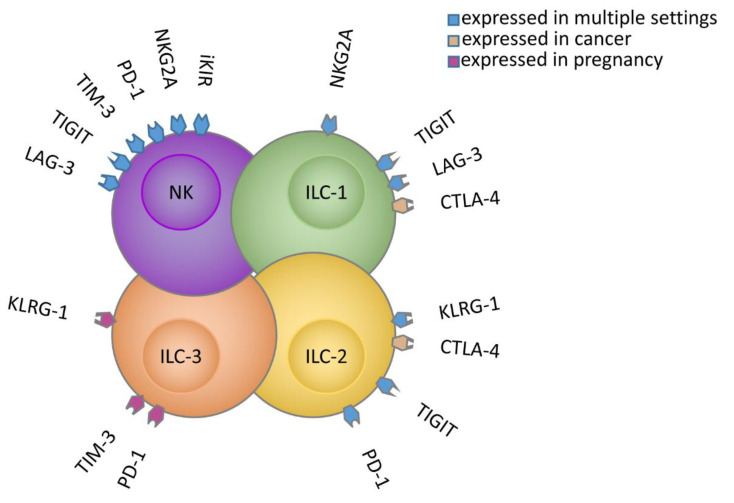
IC expression on NK cells/ILCs in healthy and pathological conditions.

Overall, we believe that, in our near future, immunotherapy protocols will need to be designed taking into account all ILCs, both cytotoxic (NK) and non-cytotoxic (helper ILCs) ones, and most importantly, ILC targeting should be tailored according to the disease (Figure 1).

## Figures and Tables

**Table 1:** Current evidence of immune checkpoint expressed in innate lymphocytes and their involvement in cancer immunotherapy.

Receptor	Cell Type	mAbs Used in Clinical Trial	Tissue/Tumor Involvement	Clinical Trial Number/Phase	Recruitment Information
PD-1	NK	Several anti-PD-1 mAbs, reviewed in [25]	Solid tumors, including Kaposi sarcoma, PC, ovarian and lung cancers [26,27,28,29,30], and HL [31]		
			Use of anti-PD-1 or anti-PD-L1 improves the anti-tumor activity of NK cells against PD-L1/2+ tumor cells [25,26,31,32]		
	ILC		Decidual ILCs during pregnancy [17]		
			Tumor-associated ILC2s (>PD-1 than circulating ILCs) [33]		
			ILC2s and ILC3s in tumors of the gastrointestinal tract (>PD-1 than in the paralesional tissue)		
			ILC3s in human malignant pleural effusion [30]		
			ILC2s in colorectal cancer model [33]		
KIRs	NK	Monotherapy with IPH2102 (lirilumab)	Solid tumors, hematologic malignancies, all pediatric tumor types [34]		
		IPH2102 combined with anti-PD-1 (nivolumab)	Bladder cancer	NCT03532451 phase 1	43 participants (active)
			SCCHN	NCT03341936 phase 2	58 participants (active/recruiting)
			Advanced solid tumors	NCT03203876 phase 1	21 participants (active)
			Leukemia	NCT02599649 phase 2	10 participants (terminated/results)
			HL, NHL, MM	NCT01592370 Phase 1/2	375 participants (active)
		IPH2102 combined with anti-PD-1 (nivolumab)+ anti-CTLA-4 (ipilimumab)	Advanced solid tumors	NCT01714739 Phase 1/2	337 participants (completed)
			Advanced solid tumors	NCT03347123 Phase 1/2	11 participants (active)
		IPH2102 combined with anti-CS1 (elotuzumab)	MM	NCT02252263 Phase 1	44 participants (completed)
		IPH2102 combined with anti-CD20 (rituximab)	CLL	NCT02481297 phase 2	7 participants (completed/results)
		Monotherapy with 1-7F9	MM	NCT00552396 phase 1	32 participants (completed/results)
		IPH2102 combined with azacytidine	AML	NCT02399917 phase 2	36 participants (terminated/results)
			MDS	NCT02599649 phase 2	10 participants (terminated/results)
NKG2A	NK	Monotherapy with IPH2201 (monalizumab) [35]	Gynecologic cancers	NCT02459301 phase 1	59 participants (completed)
		IPH2201 combined with anti-PD-L1 (durvalumab) [36]	NSCLC	NCT03822351 phase 2	189 participants (active)
			NSCLC	NCT03833440 phase2	120 participants (recruiting)
			NSCLC	NCT03794544 phase 2	80 participants (active)
			MSS-CRC	NCT04145193 phase 2	Withdrawn
			Advanced solid tumors	NCT02671435 phase 1/2	383 participants (active)
		IPH2201 combined with anti-EGFR (cetuximab) or with anti-EGFR +anti-PD-L1	SCCHN	NCT02643550 phase 1/2	140 participants (recruiting)
		IPH2201 combined with anti-Bruton’s tyrosine kinase (ibrutinib)	CLL	NCT02557516 phase 1/2	22 participants (terminated/results)
	ILC		AML [37]		
TIM-3	NK		Gastric cancer [38] Lung adenocarcinoma [39] Advanced melanoma [40] Bladder cancer [38,39,40,41]		
		Sym023	Metastatic cancer, solid tumor, lymphomas	NCT03489343 phase 1	24 participants (completed)
		Sym023 combined with anti-PD-1 or anti-LAG-3		NCT03311412 phase 1	102 participants (recruiting)
		TSR-022 combine with anti-PD-1	Advanced solid tumors	NCT02817633 phase 1	369 participants (recruiting)
		TSR-022 combine with anti-PD-1		NCT04139902 phase 2	56 participants (recruiting)
		TSR-022 combine with anti-PD-1	Primary liver cancer	NCT03680508 phase 2	42 participants (recruiting)
		BMS-986016 (relatlimab) combined with anti-PD-1 (nivolumab)	Chordoma	NCT03623854 phase 2	20 participants (recruiting)
			Metastatic uveal melanoma	NCT04552223 phase 2	27 participants (recruiting)
			Melanoma	NCT03743766 phase 2	42 participants (recruiting)
		BGB-A425 combined with anti-PD-1	Advanced or metastatic solid tumors	NCT03744468 phase 1/2	162 participants (recruiting)
		Monotherapy with MBG453	Advanced malignancies	NCT02608268 phase 1/2	252 participants (active)
			AML or high risk MDS	NCT03066648 phase 1	235 participants (recruiting)
	ILC		TIM-3+ ILC3s were found in the decidua [17]		
TIGIT	NK		Colon cancer patients and tumor models [42]		
			TIGIT blocking was suggested as a potent strategy to increase graft versus leukemia effect in alloSCT in AML [43]		
		Tiragolumab combined with anti-PD-1 (atezolizumab)	Cervical cancer	NCT04300647 phase 2	160 participants (recruiting)
			Esophageal squamous cell carcinoma	NCT04543617 phase 3	750 participants (recruiting)
			SCLC	NCT04256421 phase 3	400 participants (recruiting)
			NSCLC	NCT04513925 phase 3	800 participants (recruiting)
				NCT03563716 phase 2	135 participants (active)
			Esophageal Cancer	NCT04540211 phase 3	450 participants (recruiting)
				NCT03281369 phase 1/2	410 participants (recruiting)
			Urothelial carcinoma	NCT03869190 phase 1/2	385 participants (recruiting)
			Pancreatic adenocarcinoma	NCT03193190 phase 1/2	290 participants (recruiting)
			Advanced metastatic tumors	NCT02794571 phase 1	400 participants (recruiting)
		Tiragolumab combined with anti-VEGF (bevacizumab)	Advanced liver cancer	NCT04524871 phase 1/2	100 participants (recruiting)
		Monotherapy with BMS-986207 or combined with anti-LAG-3	MM	NCT04150965 phase 1/2	104 participants (recruiting)
		BMS-986207 combined with nivolumab	Ovarian cancer, endometrial neoplasmas, solid tumor	NCT04570839 phase 1/2	100 participants (recruiting)
		Monotherapy with OMP-313M32 or combined with anti-PD-1 (nivolumab)	Advanced and metastatic cancer	NCT03119428 phase 1	33 participants (terminated)
		Monotherapy with AB154 or combined with anti-PD-1 (zimberelimab)	Advanced solid tumors	NCT03628677 phase 1	66 participants (recruiting)
			Lung cancer	NCT04262856 phase 2	150 participants (recruiting)
	ILC		ILC1 and splenic ILC3 [44]		
LAG-3	NNK	Monotherapy with Sym022	Metastatic cancer, solid tumor, and lymphoma	NCT03489369 phase 1	15 participants (completed)
		Monotherapy with BMS-986016 (relatlimab) or combined with anti-PD-1	Brain neoplasms, glioblastoma, gliosarcoma	NCT02658981 phase 1	63 participants (active)
			Solid tumors	NCT01968109 phase 1/2	1500 participants (recruiting)
				NCT03470922 phase 2/3	700 participants (recruiting)
			SCCHN	NCT04080804 phase 2	60 participants (recruiting)
		MK-4280 combined with anti-PD-1	Solid tumors	NCT02720068 phase 1	576 participants (recruiting)
			Hematologic malignancies	NCT03598608 phase 1/2	134 participants (recruiting)
		Monotherapy with TSR-033 or combined with anti-PD-1	Advanced solid tumors	NCT03250832 phase 1	55 participants (recruiting)
		Monotherapy with LAG525 or combined with anti-PD-1	Advanced solid tumors	NCT02460224 phase 1/2	490 participants (active)
				NCT03499899 phase 2	88 participants (active)
				NCT03484923 phase 2	195 participants (recruiting)
			Solid and hematologic malignancies	NCT03365791 phase 2	76 participants (completed)
		Monotherapy with REGN3767 or combined with anti-PD-1	Advanced malignancies	NCT03005782 phase 1	669 participants (recruiting)
		Monotherapy with IMP321 (eftilagimod alpha) or combined with chemotherapy	Melanoma [45]	NCT02676869 phase 1	24 participants (completed)
			Metastatic breast cancer	NCT00349934 phase 1	33 participants (completed)
		IMP321 (eftilagimod alpha) combined with anti-PD-1	NSCLC and SCCHN	NCT03625323 phase 2	109 participants (recruiting)
	ILC		LAG-3 upregulated upon tumor conversion from NKs to ILC1s [46]		
KLRG1	ILC		KLRG1 protein expressed from ILC3s isolated from first trimester decidual tissue [17] and in a subset of ILC2s in vivo expanded with IL-25 [47]		
			KLRG1 transcripts identified in ILC2s in lung and colorectal cancer [20]		
CTLA-4	ILC		Expression of CTLA-4 in tumor associated ILC1s and ILC2s [33,46]		

AML: acute myeloid leukemia; CLL: Chronic lymphocytic leukemia; mAb: monoclonal antibody; MDS: myelodisplastic syndrome; MM: multiple myeloma; MSS-CRC: microsatellite-stable colorectal cancer; NSCLC: non-small cell lung cancer; PC: peritoneal carcinomatosis; SCCHN: squamous cell carcinoma of the head and neck; SCLC: small cell lung cancer.
